# Shikonin and 4-hydroxytamoxifen synergistically inhibit the proliferation of breast cancer cells through activating apoptosis signaling pathway in vitro and in vivo

**DOI:** 10.1186/s13020-020-00305-1

**Published:** 2020-03-10

**Authors:** Hong-Yan Lin, Hong-Wei Han, Yin-Song Wang, De-Liu He, Wen-Xue Sun, Lu Feng, Zhong-Ling Wen, Min-Kai Yang, Gui-Hua Lu, Xiao-Ming Wang, Jin-Liang Qi, Yong-Hua Yang

**Affiliations:** 1 State Key Laboratory of Pharmaceutical Biotechnology, Institute of Plant Molecular Biology, School of Life Sciences, Nanjing University, Nanjing, 210023 People’s Republic of China; 2 Co-Innovation Center for Sustainable Forestry in Southern China, Nanjing Forestry University, Nanjing, 210037 People’s Republic of China; 3 School of Life Sciences, Huaiyin Normal University, Huaian, 223300 China

**Keywords:** Breast cancer, Shikonin, 4-Hydroxytamoxifen, Drug combination, Apoptosis

## Abstract

**Background:**

Tamoxifen (TAM) is a cell type-specific anti-estrogen and is applied to improve the survival of patients with estrogen receptor positive (ER +) breast cancer. However, long-term TAM use can induce serious drug resistance, leading to breast cancer recurrence and death in patients. Further, it is almost useless among patients with estrogen receptor negative (ER −) breast cancer. Shikonin (SK) is a natural product broadly explored in cancer therapy. Some studies have demonstrated the combined treatment of SK and clinical anticancer drugs including TAM on various tumors. However, the combined effect of SK and 4-hydroxytamoxifen (4-OHT) on ER- breast cancer is not known. The current study aimed to assess the combination effects of SK and 4-OHT on human breast cancer cells, MCF-7 (ER +) and MDA-MB-435S (ER −), in vitro and in vivo and to investigate the underlying mechanisms.

**Methods:**

CCK-8 assays and flow cytometry were conducted to determine the cell viability and apoptotic profiles of human breast cancer cell lines (MCF-7 and MDA-MB-435S) treated with SK, 4-OHT, and the combination. ROS and JC-1 assays were used to determine ROS level and mitochondrial membrane potential. Western blot analysis was performed to investigate proteins that are associated with apoptosis. Haematoxylin & Eosin (HE) staining was used to detect the tumor and kidney morphology of mice. TUNEL and immunohistochemical staining were performed to detect Ki67 expression level and cell apoptotic profile in tumor tissues.

**Results:**

SK and 4-OHT synergistically inhibited MCF-7 and MDA-MB-435S cell proliferation and promoted apoptosis by reducing mitochondrial membrane potential and increasing the intracellular ROS level. The combination of SK and 4-OHT activated the mitochondrial-dependent apoptosis and the death receptor pathways, significantly regulating the PI3K/AKT/Caspase 9 signaling pathway. Compared with SK and 4-OHT alone, the combination of SK and 4-OHT could better inhibit tumor growth in mice.

**Conclusion:**

The combination of SK and 4-OHT shows highly efficient anticancer effects on breast cancer therapy. SK may be a promising candidate as an adjuvant to 4-OHT for breast cancer treatments, especially for ER- breast cancer.

## Background

Breast cancer is the most commonly diagnosed cancer and the leading cause of cancer death among females worldwide [1]. Breast cancer is treated using various therapies, including surgery [2], radiotherapy [3], endocrine therapy for patients with estrogen receptor positive (ER +) breast cancer [4], and targeted therapy, such as monoclonal antibody trastuzumab targeting HER-2 [5], and chemotherapy which is an important strategy for patients diagnosed with advanced stage cancer [6]. Tamoxifen (TAM), which functions as a cell type-specific anti-estrogen, is the dominant endocrine treatment of breast cancer with demonstrated efficacy for over four decades. TAM is applied to improve the survival of patients with ER + breast cancer [7]. However, long-term TAM use can induce drug resistance, leading to breast cancer recurrence and death [8]. TAM is almost useless among patients with estrogen receptor negative (ER −) breast cancer, thereby limiting its application to breast cancer treatment [9].

Combined therapy is considered as an excellent treatment strategy. Many studies aimed to restore TAM response in ER + breast cancer [10–14] and increase TAM’s sensitivity in patients with ER − breast cancer through combined therapy [15–17]. The limitation of TAM can be addressed by searching for effective chemosensitizers that augment TAM’s efficiency and overcome multidrug resistance.

Shikonin (SK) is a natural naphthoquinone compound extracted from the roots of the Chinese herbal plant *Lithospermum erythrorhizon*, exhibiting powerful anticancer activities on various breast cancer cells [18]. This effect is accomplished through multiple pathways that disrupt ER recruitment [19], induce programmed cell death [20, 21], and inhibit metalloprotease (MMP)-9 [22] and topoisomerase [23]. SK can also target tumor-specific pyruvate kinase-M2 (PKM2) and induce necroptosis [24], thereby enabling SK to avoid drug resistance. SK showed similar toxicity toward drug-sensitive and drug-resistant breast cancer cells [25]. The combined treatment of SK and clinical anticancer drugs has been widely investigated. For instance, SK can effectively sensitize A549 cells to TRIAL-induced cytotoxicity through the modulation of the JNK, STAT3 and AKT pathways [26]. In turn, TRIAL can also enhance SK induced apoptosis through reactive oxygen species (ROS)/JNK signaling in cholangiocarcinoma cells [27]. SK can be used as a synergistic agent to cisplatin to achieve excellent anticancer activity by inducing intracellular oxidative stress in colon cancer cells [28]. The combination of SK and taxol can also reverse multidrug resistance in human ovarian cancer A2780 cells in a P-glycoprotein-independent manner through enhanced ROS generation [29]. For human breast cancer cells, SK can sensitize MDA-MB-231 cell to chemotherapy by taxol through the activation of the ERKs and AKT pathways, improve mice survival and inhibit tumor growth in mice [30, 31]. A previous work showed that SK could reduce TAM-resistance in MCF-7R cells by targeting IncRNA uc.57 and its downstream gene BCL11A [32]. Although the combination of SK and TAM was not an unprecedented strategy for ER + breast cancer therapy, their combined effects on ER- breast cancer were notknown. SK and its derivatives belong to a class of necroptotic or apoptotic inducers. They can bypass anticancer drug resistance [24, 25]. Therefore, the adjuvant activity of SK to 4-hydroxytamoxifen (4-OHT) on the inhibition of ER + and ERT − human breast cancer cell proliferation in vitro and in vivo was investigated in this work, and the mechanisms of the combination action in terms of cell apoptosis were explored.

## Materials and methods

### Cell lines and culture conditions

The human breast cancer cell lines (MCF-7 and MDA-MB-435S) were originally obtained from the American Type Culture Collection (Manassas, VA, USA). Cells were cultured in DMEM (Gibco, USA) containing 10% fetal bovine serum (Gibco, USA) supplemented with 1% penicillin–streptomycin (Gibco, USA) and maintained at 37 °C in a humidified atmosphere of 95% air and 5% CO_2_ incubator.

### Reagents and antibodies

Cell Counting Kit-8 (CCK-8, #40203ES92) and 4-hydroxytamoxifen (4-OHT, HY16950) were purchased from MedChemExpress (MCE, USA). Shikonin (SK) was prepared by our laboratory and its structure characteristic data were shown in *SI Appendix*. SK and 4-OHT were dissolved in dimethyl sulfoxide (DMSO, D2650, Sigma, USA). The maximum final concentration of 0.1% DMSO was used as a control. The antibodies were purchased from proteintech group (USA).

### In vitro cytotoxic assay

The in vitro cytotoxic effect of SK and 4-OHT was determined by using CCK-8 assay kit. Cells were seeded at the density of 5 × 10^3^ per well into 96-well plated and maintained at 37 °C, 5% CO_2_ atmosphere overnight. Cells were treated with either SK, or 4-OHT, or the combination, and continue to be incubated for 24 h. Then, the supernatant was discarded and fresh medium (100 μL) was added into the 96-well plated. Meanwhile, CCK-8 solution (10 μL) was added to each well, after which the cells were further incubated for 2 h. The absorbance was measured and recorded on an ELISA reader (ELx800, BioTek, USA) at a test wavelength of 450 nm. In all experiments, three replicate wells were set for each drug concentration and all tests were performed at least three times.

To evaluate the synergistic efficacies of SK and 4-OHT, the Q value method of was used to evaluate the interaction between SK and 4-OHT on MCF-7 cells according to the equation Q = E_a+b_/(E_a_ + E_b_−E_a_ × E_b_), where E_a+b_ is the synergism inhibition rate, E_a_ and E_b_ is the inhibition rate of drug A and drug B used alone, respectively. Synergism of the two drugs defined as a significantly greater effect when used in combination than the sum of the effects of the two drugs used alone. Q < 0.85 suggests antagonism between the two drugs, 0.85 ≤ Q<1.15 addition of their effect, and Q ≥ 1.15 synergism between them.

### Cell apoptosis assay

Cell apoptosis were examined using Annexin V-FITC/PI dual staining detection kits (#40203ES60) which were purchased from YEASEN (Shanghai, China). Briefly, MCF-7 and MDA-MB-435S cells were stained with Annexin V-FITC and PI and then monitored for apoptosis by flow cytometry. Specifically, cells were seeded at the density of 5 × 10^4^ per well into 6-well plated and maintained at 37 °C, 5% CO_2_ atmosphere overnight. Then, cells were treated with SK, 4-OHT, or the combination for 24 h, after which the cells were collected and washed twice with pre-cooling phosphate-buffered saline (PBS) and stained with 5 µL of Annexin V and 10 µL of PI (5 µg/mL) in 100 μL of 1 × binding buffer (10 mM HEPES, pH 7.4, 140 mM NaOH, 2.5 mM CaCl_2_) for 15 min at room temperature in the dark. Apoptotic cells were quantified using BD FACScalibur Flow Cytometer (BD, USA). Data were analyzed using Flowjo 7.6.1 software. Both early apoptotic (Annexin V + and PI−) and late apoptotic (Annexin V + and PI +) cells were detected.

### Mitochondrial membrane potential (∆ѱm) analysis

Mitochondrial transmembrane potentials were detected by using a Mitochondrial Membrane Potential Assay Kit (#C2006, Beyotime Institute of Biotechnology, Haimen, China) with JC-1 dye (lipophilic cation 5, 5′, 6, 6′-tetrachloro-1, 1′, 3, 3′-tetraethylbenzimidazolcarbocyanine iodide). Briefly, cells were seeded into 6-well plates at 5 × 10^4^ cells per well and maintained at 37 °C, 5% CO_2_ atmosphere overnight. After treated with SK, 4-OHT, or the combination for 24 h, the cells were collected and washed twice with ice-cold PBS. Then cells were re-suspension with 1 × JC-1 staining buffer and incubated in CO_2_ incubator for 15–30 min. Finally, cells were washed twice with PBS and re-suspension with 500 μL PBS before being analyzed by BD FACScalibur Flow Cytometer (BD, USA).

### Intracellular reactive oxygen species (ROS) levels detection by flow cytometry

Intracellular reactive oxygen species (ROS) levels were measured by ROS Assay Kit (#S0033, Beyotime Institute of Biotechnology, Haimen, China) with an oxidation-sensitive fluorescent probe dye, DCFH-DA (2, 7- dichlorodihydro fluorescein diacetate). In brief, cells were seeded in 6-well plates (5 × 10^3^ cells per well) and incubated at 37 °C, 5% CO_2_ atmosphere for 24 h. Exponentially growing cells were then incubated with 3 μM SK, 17.5 μM 4-OHT, or the combination for 12 h, after which the cells were collected and washed with PBS, and then incubated at 37 °C with DMEM containing 10 μM DCFH-DA for 30 min. After that, cells were washed three times using serum-free DMEM. The samples were finally analyzed using a FACScan flow cytometer and Flowjo 7.6.1 software.

### Protein extraction and western boltting

Total proteins were extracted from human breast cancer cells after treated with RIPA lysis buffer containing 1% PMSF and centrifuged at 16,000*g* at 4 °C for 15 min using Eppendorf 5810R centrifuge. Protein concentrations were measured using the BCA Protein Assay kit (#23227 Thermo Fisher Scientific, USA). Approximately 80 µg of total protein were separated by 10% SEMS–polyacrylamide gels and transferred to PVDF membranes. After blocked with 5% defatted milk solution, membranes were incubated with primary antibodies, such as Smac/Diablo (#10434-1-AP, rabbit polyclonal IgG, 1: 1000 dilution); PI3K (#13329-1-AP, rabbit polyclonal IgG, 1: 1000 dilution), AKT (#10176-2-AP, rabbit polyclonal IgG, 1: 1000 dilution), Caspase 9 (#10380-1-AP, rabbit polyclonal IgG, 1: 1000 dilution), PARP-1 (#66520-1-Ig, mouse monoclonal IgG, 1: 600 dilution), Bad (#10435-1-AP, rabbit polyclonal IgG, 1: 1000 dilution), Bcl-2 (#12789-1-AP, rabbit polyclonal IgG, 1:1000 dilution), Bax (#50599-2-Ig, rabbit polyclonal IgG, 1:1000 dilution), Caspase 8 (#13423-1-AP, rabbit polyclonal IgG, 1: 1000 dilution), Fas (#13098-1-AP, rabbit polyclonal IgG, 1:1000 dilution), Bid (#10988-1-AP, rabbit polyclonal IgG, 1: 1000 dilution), Caspase-3 (#19677-1-AP, rabbit polyclonal IgG, 1:1000 dilution) and GAPDH (#10494-1-AP, rabbit polyclonal IgG, 1: 1000 dilution) at 4 °C overnight. After thrice washing in TBST for each 5 min, membranes were incubated with HRP-conjugated secondary antibodies (#SA00001-1, #SA00001-2, 1: 5000 dilution) at room temperature for 2 h. Detection was performed by an enhanced chemiluminescent reagent (Thermo Fisher Scientific, USA) according to the manufacturer’s instructions. Bands were then recorded by a digital camera (Tanon 5200, Shanghai, China). Finally, the results were analyzed with Image J Software (National Institutes of Health, BetheSEMa, Maryland, USA), and all the targeted proteins were normalized to GAPDH.

### Animals and treatment

Female nude mice at 6–8 weeks old were purchased from Model Animal Research Center of Nanjing University (Nanjing, China). They were kept at 22 °C–24 °C with a 12 h light/12 h dark cycle in a pathogen-free isolation facility. They were allowed to adapt for 1 week prior to experimentation. Cultured MCF-7 cells were washed and re-suspended in ice-cold PBS. Portions of the suspension (6 × 10^6^ cells in 0.1 mL) were subcutaneously injected into the right flanks of each mouse. After 2 weeks, the mice bearing tumors (100 mm^3^ on average) were randomly grouped into four (n = 8 mice per group) in accordance with tumor volumes. SK (1.5 mg/kg) and 4-OHT (3 mg/kg) were dissolved in DMSO and administered once every 2 days for six times via intraperitoneal injection. The vehicle (DMSO)-treated group was included as a control. The body weight and tumor volumes were measured and recorded every 2 days. The long (*L*) and short (*S*) diameters of a tumor were measured with a Vernier caliper, and the tumor volume was calculated using the following formula: *L* × (*S*)^2^/2. On the 15th day, all the mice were euthanized. The tumors were separated, and their weights were determined. The tumor growth inhibition rate was calculated using the following formula: (1−*W*_*1*_/*W*_*2*_) *100%, where *W*_*1*_ is the tumor wet weight of drug group, and *W*_*2*_ is the tumor wet weight of the control group. All animal experiments were conducted in strict compliance with the Guidelines for the Care and Use of Laboratory Animals of Nanjing University and approved by the Laboratory Animal Ethics Committee of School of Life Sciences, Nanjing University.

### Haematoxylin & Eosin (HE) staining

The tumor and kidney tissues obtained from mice were fixed in 4% papaformaldehyde for over 12 h and were embedded in paraffin. Then, optimum cutting temperature packages tissues to slice as 20–30 μm sections. Then, sections were stained with haematoxylin and eosin.

### Immunohistochemical analysis

Immunohistochemistry was performed for Anti-Ki67. The tissues were incubated with primary antibody (ab15580, Abcam, USA) at 4 °C overnight after deparaffinized and rehydrated. The Alexa Fluor 488 labeled anti-rabbit secondary antibody (ab150077, Abcam, USA) were treated 1 h at room temperature. Finally, signals were developed with Hematoxylin and DAB (Dako, Agilent Technologies, USA).

### TUNEL staining analysis

Cell apoptosis of tumor tissues were performed by using TUNEL Apoptosis Assay Kit (TUN11684817, Roche, Swiss) according to the instructions.

### Data and statistical analysis

All experiments were repeated at least three times. Data are presented as mean ± S.E.M. and statistical comparisons among groups of controls, individual agents, and combinations of agents using one-way ANOVA using GraphPad PRISM5 (Graphpad Inc., La Jolla, USA). *P < 0.05 was considered as statistically significant; **P < 0.01 was considered as statistically very significant.

## Results

### Combination of SK and 4-OHT exhibited synergistic or enhanced anticancer effects on MCF-7 and MDA-MB-435S

**Fig. 1:**
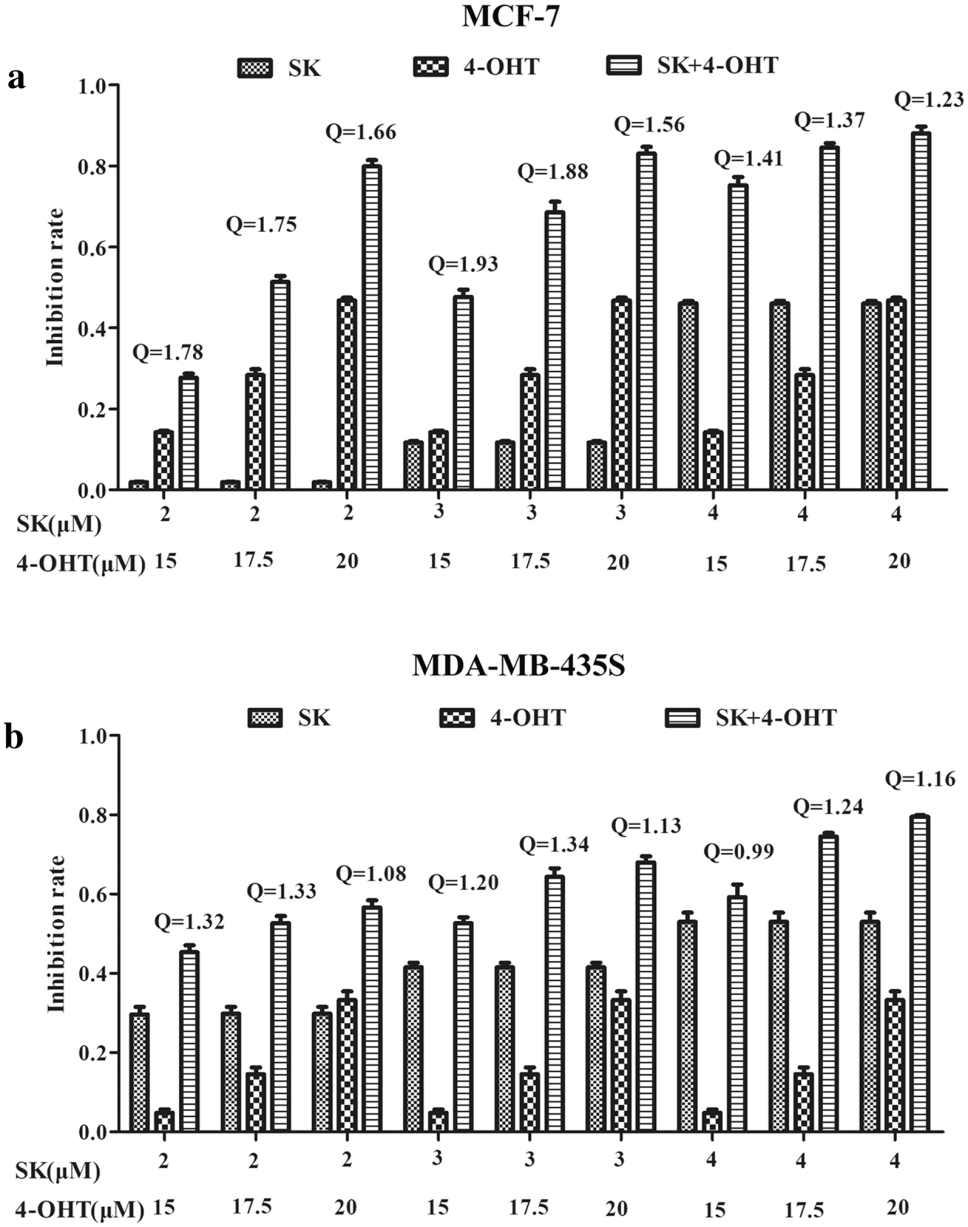
Effects of SK, 4-OHT, and the combination on cell proliferation. **a** The proliferation inhibition rate of MCF-7 cells treated with SK (2, 3, 4 μM), 4-OHT (15, 17.5, 20 μM), or the combination at 24 h. Q < 0.85 suggests antagonism between the two drugs, 0.85 ≤ Q<1.15 addition of their effect, and Q ≥ 1.15 synergism between them. **b** The proliferation inhibition rate of MDA-MB-435S cells treated with SK (2, 3, 4 μM), 4-OHT (15, 17.5, 20 μM), or the combination at 24 h. Data are representative of three independent experiments. Each value represents the mean ± S.E.M (n = 3)

CCK-8 assay was used to evaluate the antiproliferation effects of SK, 4-OHT, and their combination on the two types of breast cancer cell lines, MCF-7 (ER +) and MDA-MB-435S (ER−). The combination effects of SK and 4-OHT were evaluated by calculating the Q values. The results shown in Fig. 1 indicates that the effects of all combination groups on MCF-7 cell line were synergistic (Q > 1.15), whereas only two-thirds of the combination groups showed synergistic effects on MDA-MB-435S cell line. The other three combinations showed enhanced anticancer effects (1.15 > Q>0.85). By comparing the Q values shown in Additional file 1: Table S1, we found that the combination of 3 μM SK and 15 μM 4-OHT showed the strongest synergistic effect on MCF-7 cell line (Q = 1.93). The combination significantly inhibited the cell viability with an inhibition rate of 47%. The inhibition rates of single drug SK (3 μM) and 4-OHT (15 μM) were 12% and 14%, respectively. For MDA-MB-435S cell line, the best synergistic combination was 3 μM SK and 17.5 μM 4-OHT (Q = 1.34). The inhibition rate of such a combination reached 66%, whereas the inhibition rates of single drug SK (3 μM) and 4-OHT (17.5 μM) were 41% and 14%, respectively (Additional file 1: Table S2). Therefore, such combinations were the most optimal treatments and were subsequently used in the in vitro experiments.

### Combination of SK and 4-OHT induced cell apoptosis in MCF-7 and MDA-MB-435S

**Fig. 2:**
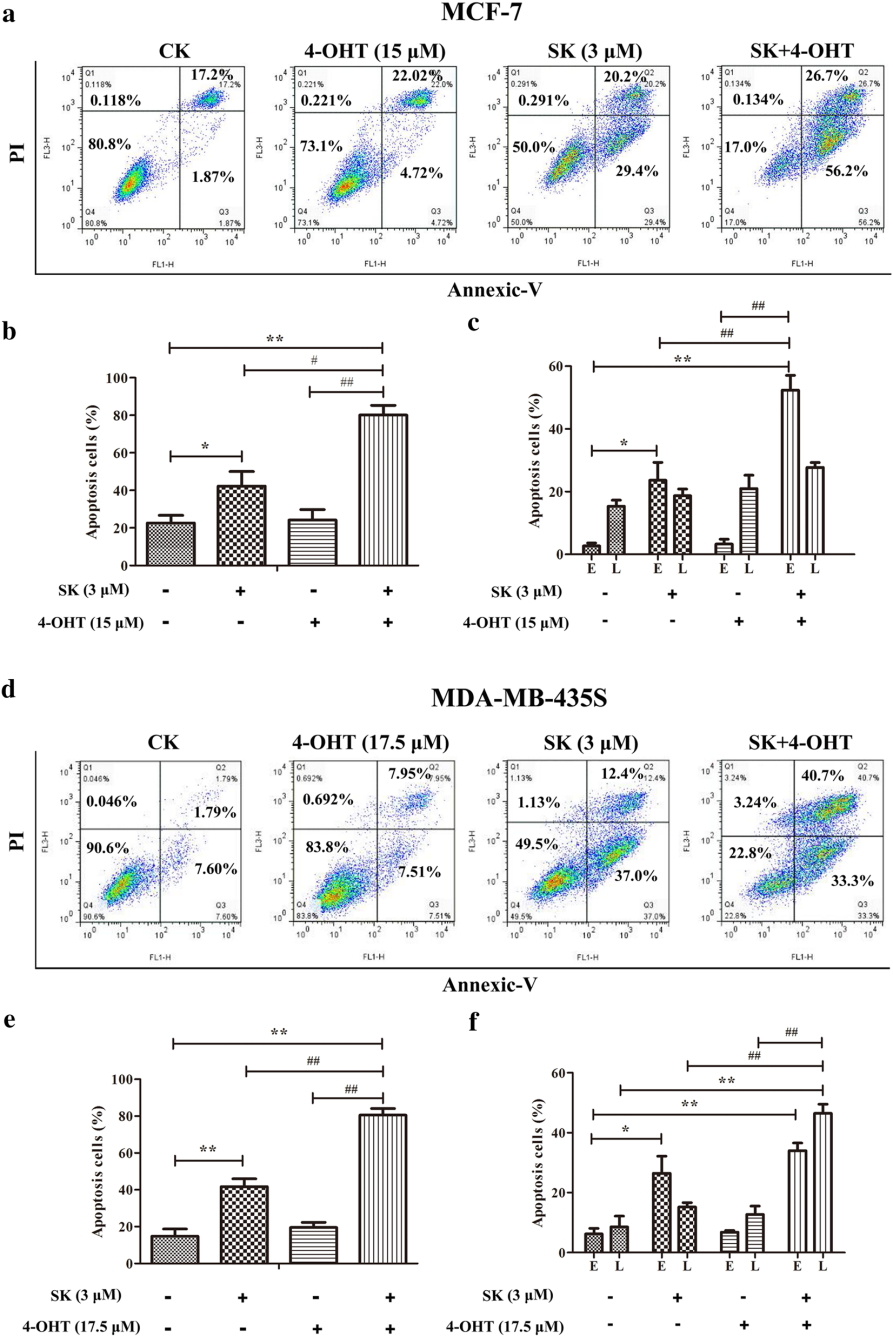
Effects of SK, 4-OHT, and the combination on cell apoptosis. **a** Representative dot-plots from cytometrically illustrating apoptotic status in MCF-7 cells. **b** Statistic analysis of apoptotic cells (percentage of cells in Q2 and Q3). **c** Statistic analysis of early (E, percentage of cells in Q3) and late (L, percentage of cells in Q2) apoptotic cells. **d** Representative dot-plots from cytometrically illustrating apoptotic status in MDA-MB-435S cells. **e** Statistic analysis of apoptotic cells (percentage of cells in Q2 and Q3). **f** Statistic analysis of early (E, percentage of cells in Q3) and late (L, percentage of cells in Q2) apoptotic cells. Images are representative of three independent experiments. Data are mean ± S.E.M. from three independent experiments (*P < 0.05, **P < 0.01 compared with the negative control; ^##^*P* < 0.01 compared with the combination group)

Annexin V/propidium iodide (PI) double staining results demonstrated that the combination of SK and 4-OHT could induce cell apoptosis more effectively than the cells that were only treated with SK or 4-OHT in MCF-7 and MDA-MB-435S (Fig. 2). Further analysis indicated that the combination significantly increased early apoptotic cells in the MCF-7 cell line (Fig. 2c) and late-apoptotic cells in MDA-MB-435S cell line (Fig. 2f).

### Combination of SK and 4-OHT decreased the mitochondrial membrane potential in MCF-7 and MDA-MB-435S

**Fig. 3:**
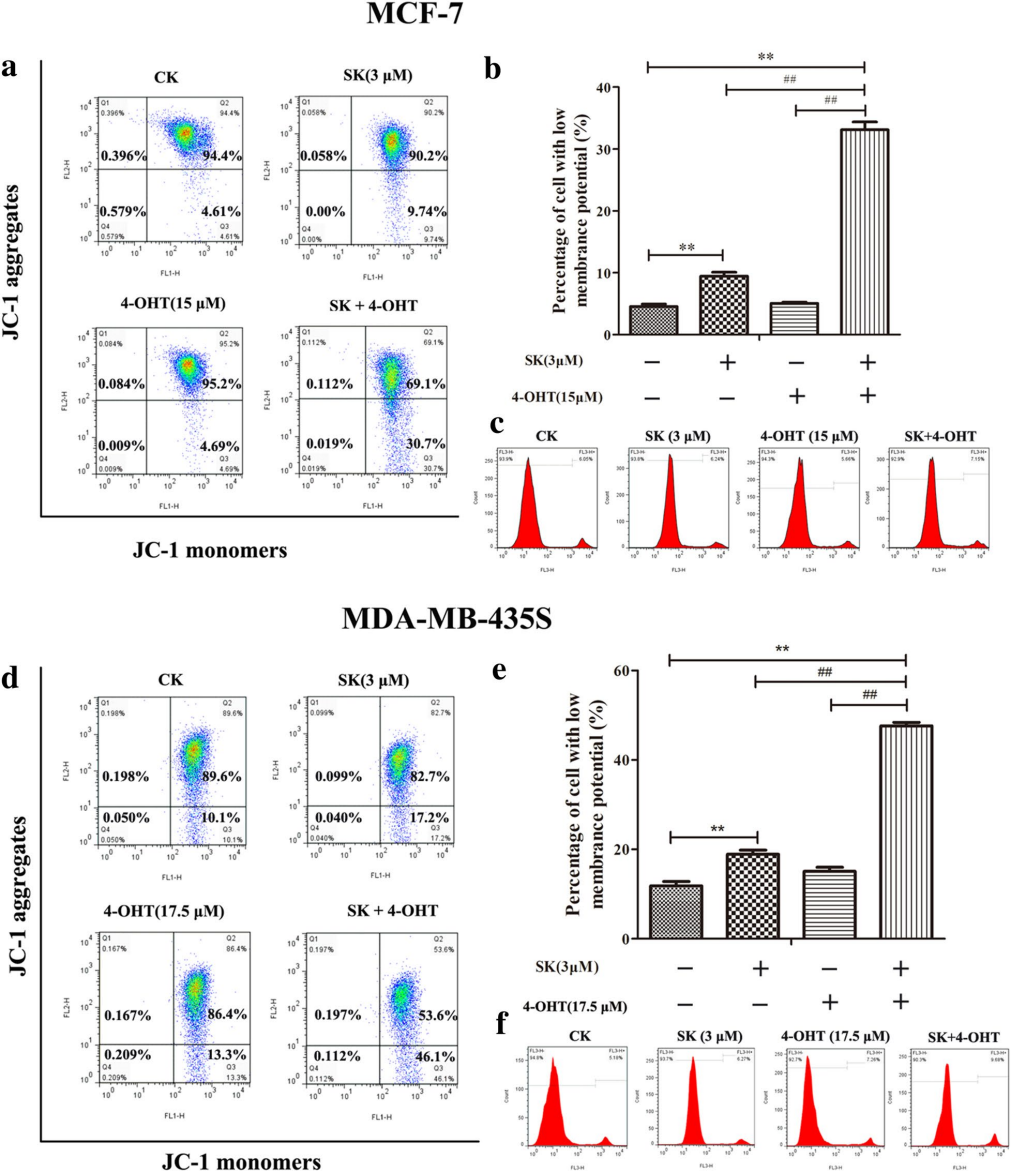
Effects of SK, 4-OHT, and the combination on decreasing the mitochondrial membrane potential of cells. **a** Flow cytometry analysis of MCF-7 cells with high Δ*ѱ*_*m*_ (%) by JC-1 staining. **b** Statistic analysis of MCF-7 cells with low Δ*ѱ*_*m*_ (%) (percentage of cells in Q3). **c** The representative image of PI staining of MCF-7 cells. **d** Flow cytometry analysis of MDA-MB-435S cells with high Δ*ѱ*_*m*_ (%) by JC-1 staining. **e** Statistic analysis of MDA-MB-435S cells with low Δ*ѱ*_*m*_ (%) (percentage of cells in Q3). **f** The representative image of PI staining of MDA-MB-435S. Data are mean ± S.E.M. from three independent experiments (**P < 0.01 compared with the negative control; ^##^*P* < 0.01 compared with the combination group)

The mitochondrial membrane potential is an important parameter that regulates mitochondrial functionality and cellular processes. Its dissipation may initiate apoptosis [33]. As shown in Fig. 3a, SK and the combination treatment could decrease the mitochondrial membrane potential in MCF-7 cells but not 4-OHT. Moreover, compared with SK (high *ΔΨm* 90.2%) and 4-OHT (high *ΔΨm* 95.2%), the combination (high *ΔΨm* 69.1%) treatment caused a more serious loss of membrane potential (Fig. 3b). The PI staining results in Fig. 3c show that the above drug concentrations and treatment times could not cause false positives by inducing late apoptosis. The reliability of mitochondrial membrane potential results was ensured. Meanwhile, a similar result was observed in the MDA-MB-435S cell line. The combination treatment resulted in the lowest mitochondrial membrane potential among the treatments, as follows: combination treatment, 53.6%; SK, 82.7%; and 4-OHT, 86.4% (Fig. 3d, e).

### Combination of SK and 4-OHT increased intracellular ROS level in MCF-7 and MDA-MB-435S

**Fig. 4:**
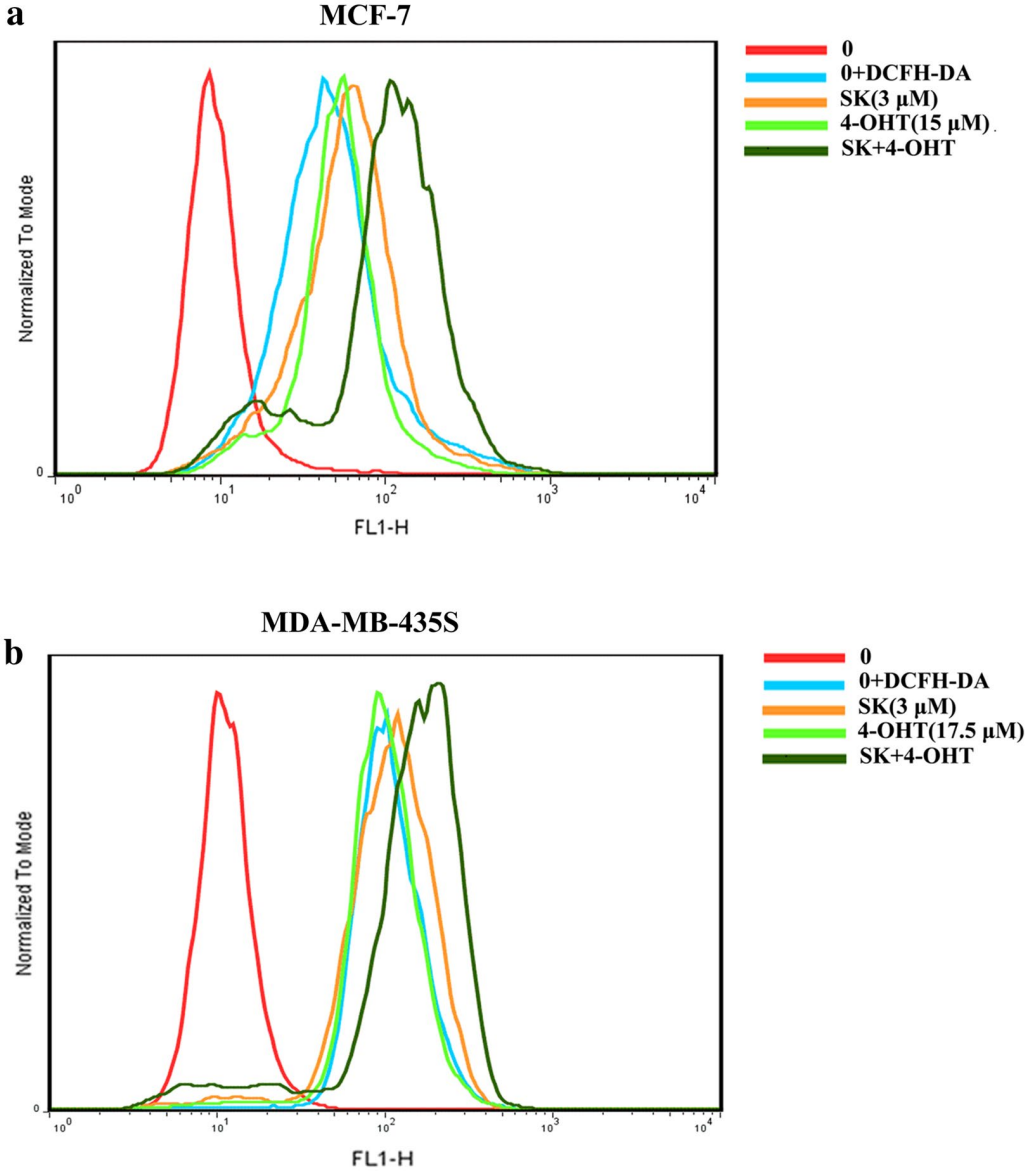
Effects of SK, 4-OHT, and the combination on the ROS production in cancer cells. **a** MCF-7 cell; **b** MDA-MB-435S cell. The image shown is the most representative result of three replicates

The effects of SK and 4-OHT alone or combination on intracellular ROS level was determined by flow cytometry in MCF-7 and MDA-MB-435S cell lines. The combination group induced the generation of ROS more effectively than SK and 4-OHT in MCF-7 and MDA-MB-435S cells (Fig. 4).

### SK and 4-OHT combination regulated the expression level of apoptotic-related proteins

**Fig. 5:**
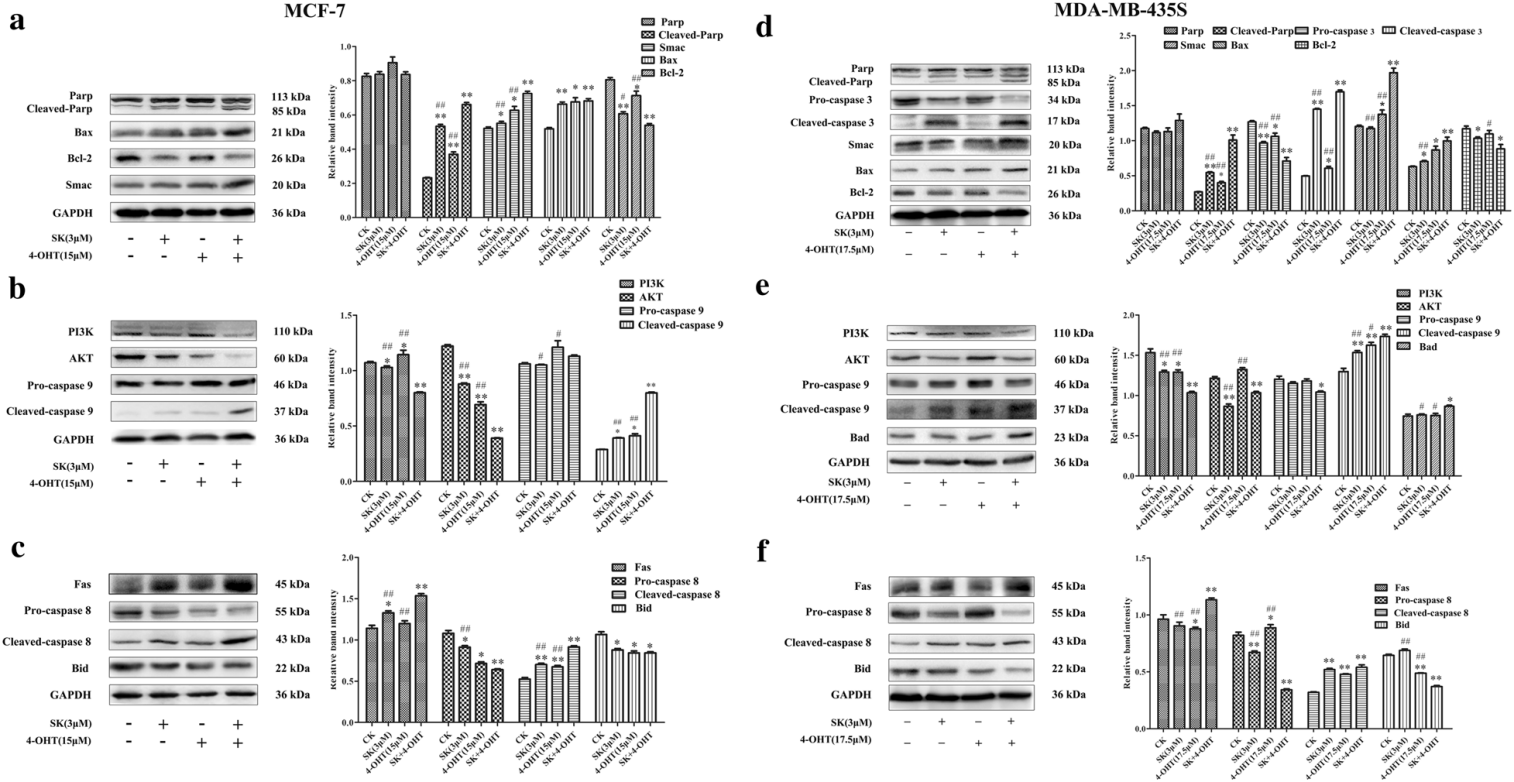
Western blot analysis of apoptotic proteins. **a** Effect of SK (3 μM) and 4-OHT (15 μM) alone or in combination on the expression of mitochondria-dependent apoptosis related proteins of MCF-7 cells. **b** Western blot result of proteins related to PI3K/AKT/Caspase 9 pathway of MCF-7 treated with SK (3 μM) and 4-OHT (15 μM) alone or in combination. **c** Western blot result of proteins related to cell death receptor pathway of MCF-7 treated with SK (3 μM) and 4-OHT (15 μM) alone or in combination. **d** Western blot result of ERα expression of MCF-7 treated with SK (3 μM) and 4-OHT (17.5 μM) alone or in combination. **e** Effect of SK (3 μM) and 4-OHT (17.5 μM) alone or in combination on the expression of mitochondria-dependent apoptosis related proteins of MDA-MB-435S cells. **f** Western blot result of proteins related to PI3K/AKT/Caspase 9 pathway of MDA-MB-435S treated with SK (3 μM) and 4-OHT (17.5 μM) alone or in combination. **g** Western blot result of proteins related to cell death receptor pathway of MCF-7 treated with SK (3 μM) and 4-OHT (17.5 μM) alone or in combination separated by SDS-PAGE. Relative band intensity was determined by Image J software. GAPDH served as a loading control. Images are representative of three independent experiments. Data are mean ± S.E.M. from three independent experiments (*P < 0.05, **P < 0.01 compared with the negative control; ^#^*P* < 0.05, ^##^*P* < 0.01 compared with the combination group)

The effect of SK combined with 4-OHT on the expression of apoptosis-related proteins were studied using Western blot assay. The mitochondrial apoptosis pathway and the death receptor pathway were activated in MCF-7 and MDA-MB-435S after the combination treatment (Fig. 5). In the mitochondria-dependent apoptosis pathway, both cells showed increased expressions of Cleaved PARP and pro-apoptotic factor Smac, but the expression of the anti-apoptotic protein Bcl-2 was downregulated. The combination also increased the expression of Bax and Cleaved-caspase 3 in MDA-MB-435S (Fig. 5a, d). The key proteins in the PI3K-AKT-Caspase 9 pathway were detected, and the expressions of PI3K in MCF-7 and MDA-MB-435S cells were significantly reduced after the combination treatment (Fig. 5b, e). However, AKT expression was markedly decreased in MCF-7, but not significantly in MDA-MB-435S. The combination treatment increased the expression of Cleaved caspase 9 in the two cell lines. For the death receptor pathway, the combined group showed high Fas protein and Cleaved-caspase 8 expression but low downstream Bid protein expression compared with the SK and 4-OHT groups (Fig. 5c, f). The above results elucidated the molecular mechanism of the SK and 4-OHT synergistic effects on MCF-7 and MDA-MB-435S in terms of apoptotic pathways.

### Combined therapy suppressed tumor growth in vivo

**Fig. 6:**
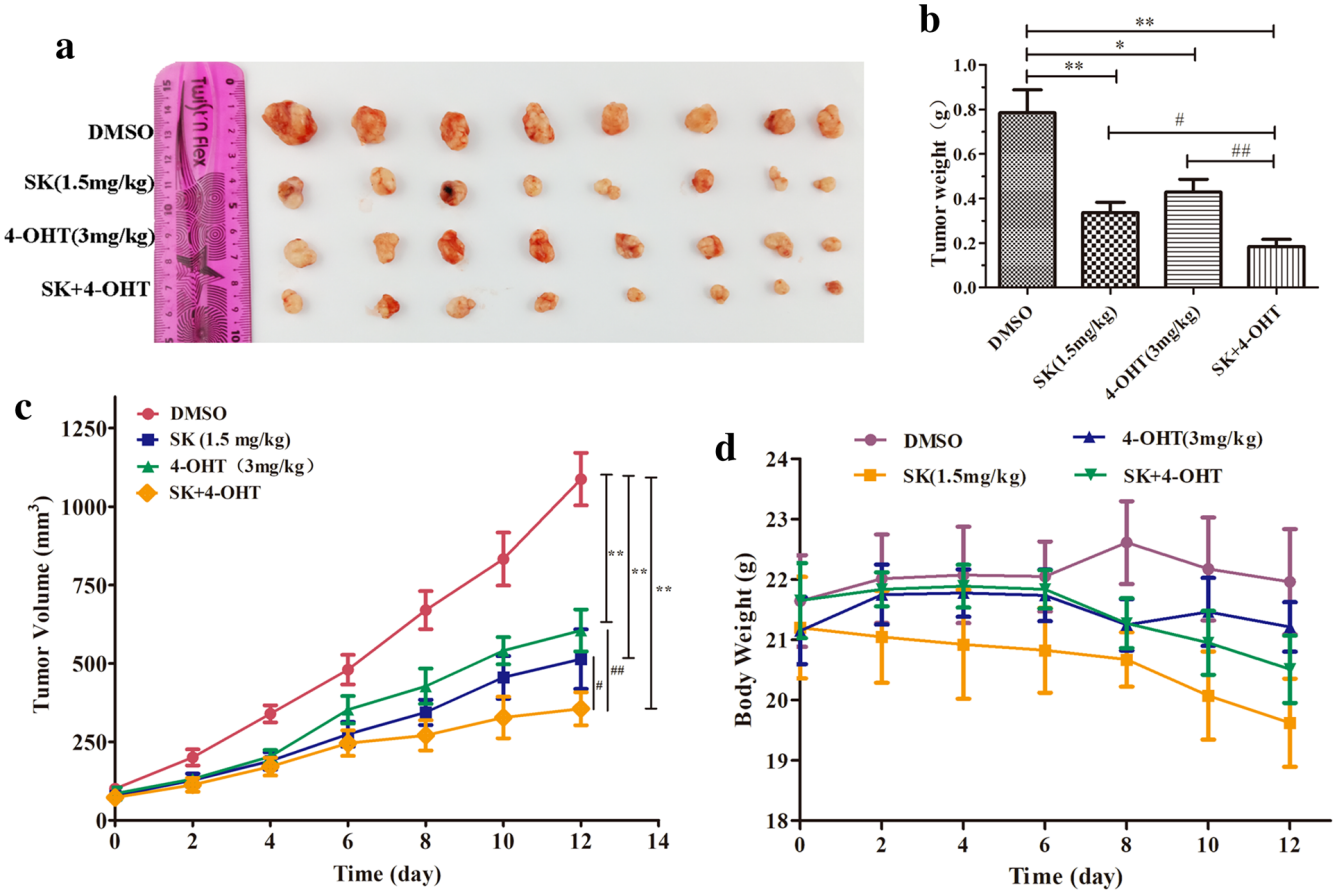
Effects of SK, 4-OHT, and the combination on tumor growth in vivo. **a** Representative image of tumors from the implanted mice. **b** Tumor weight. **c** Tumor volumes. **d** Mice body weight. Data are the mean ± S.E.M. of 8 mice per group. (*P < 0.05, **P < 0.01 compared with the vehicle (DMSO); ^#^*P* < 0.05, ^##^*P* < 0.01 compared with the combination group)

**Fig. 7:**
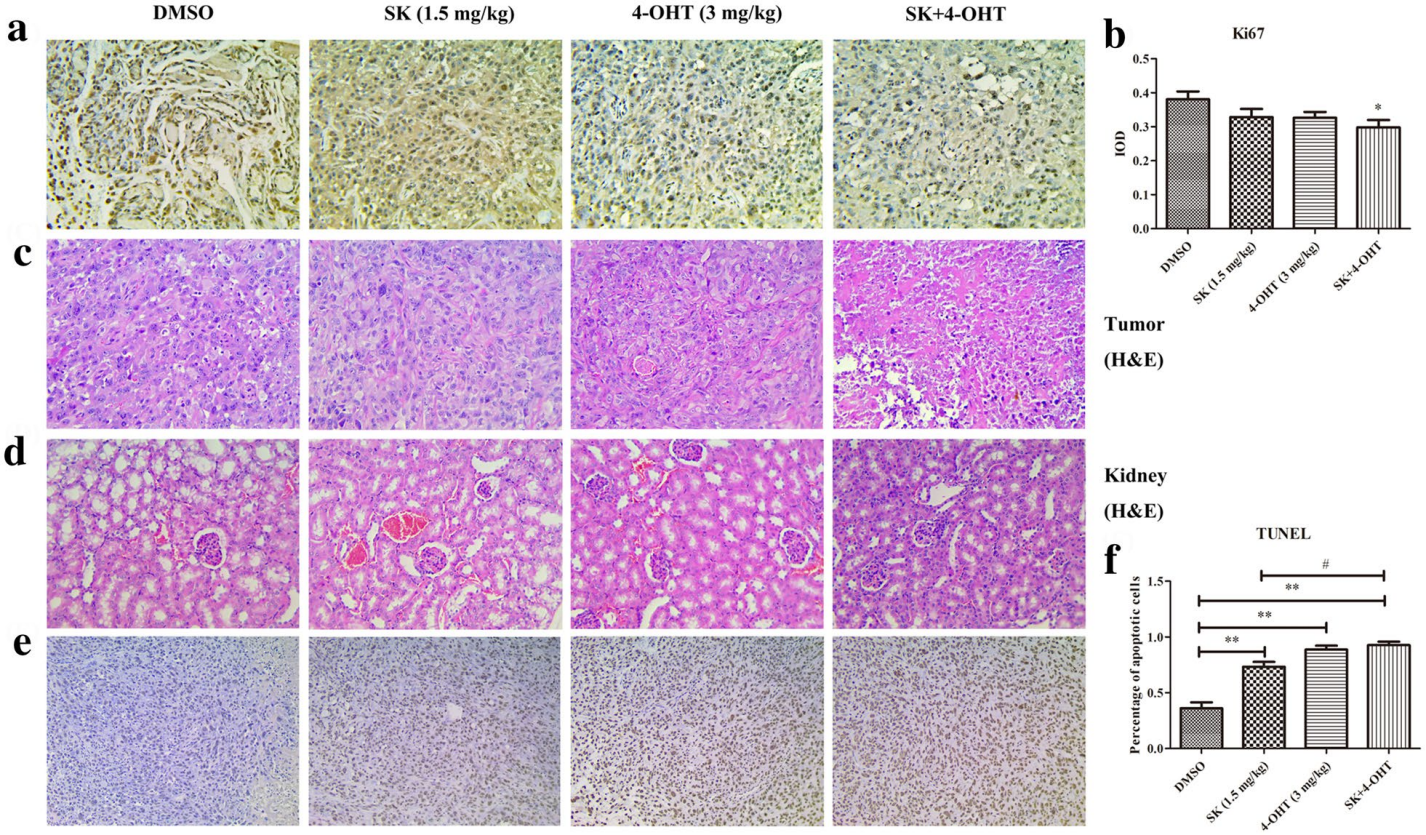
Effects of SK, 4-OHT, and the combination on tumor tissues and kidney in vivo. **a** Ki67 expression in tumors of each group detected by immunohistochemistry. Original magnification, × 200. **b** The IOD of Ki67 positive cells was calculated. **c** H&E staining of tumor tissues of each group. Original magnification, × 200. **d** H&E staining of kidney of each group. Original magnification, × 200. **e** Tumor cells of each group underwent apoptosis (TUNEL). Original magnification, × 100. **f** Statistic analysis of cells underwent apoptosis. Data are the mean ± S.E.M. of three replicates. (*P < 0.05, **P < 0.01 compared with the vehicle (DMSO); ^#^*P* < 0.05, ^##^*P* < 0.01 compared with the combination group)

Female BALB/c mice were subcutaneously inoculated with MCF-7 cells to form sizeable tumors and to determine the in vivo safety and efficiency of the combined treatment. The mice were randomly grouped into four, and each group was treated with the vehicle (DMSO), SK (1.5 mg/kg), 4-OHT (3 mg/kg), and their combination, respectively. As shown in Fig. 6a, c, SK and 4-OHT monotherapy could effectively inhibit the growth of transplanted tumor in nude mice compared with the control group. The combination group had the smallest mean tumor volume. Furthermore, the mean tumor weight of the combination group was 0.18 g, which was 58% lower than that of 4-OHT and 47% lower than that of SK (Fig. 6b). In vivo, the tumor growth inhibition rate of the combination was 76.65%, which was higher than those of SK (57.20%) and 4-OHT (45.44%) independent treatments (Additional file 1: Table S3). In addition, 4-OHT and the combination did not significantly reduce the mice body weight. By contrast, SK had certain side effects on mice and had a slight effect on their body weight (Fig. 6d). Further immunohistochemical analysis and H&E staining results of tumor tissues indicated that the combination could inhibit tumor cell more effectively than SK or 4-OHT (Fig. 7a–c). The combination treatment resulted in less severe renal injury compared with SK (Fig. 7d) and induced more significant apoptosis in tumor tissues than SK or 4-OHT monotherapies (Fig. 7e, f).

## Discussion

SK is a well-known active ingredient isolated from the root of a Chinese herbal medicine that has been used for thousands of years. Its extensive and remarkable antitumor activities, including its anti-breast [18], gastric [34], colon [35], glioma [36] effects, have attracted considerable attention. Breast cancer research findings show SK could inhibit estrogen-dependent tumor cell growth and promote the anti-estrogen effect of TAM, restoring its efficacy against cancer cells [19]. In addition, SK could bypass cancer drug resistance by targeting the “weak point” of apoptotic/drug resistant cancers [24, 25]. This “weak point” is necroptosis, a basic cell-death pathway distinct from apoptosis. SK can induce apoptosis and necroptosis in drug-sensitive and resistant cancer cell lines by targeting PKM2, which is universally expressed in cancer cells and controls the last-limiting step of glycolysis [37]. SK can also modulate the PI3K/AKT pathway to suppress the growth and survival of cancer cells [32]. The overexpression of PI3K/AKT is closely related to TAM resistance [38]. Thus, SK might be beneficial for the prevention of ER + breast cancer cell resistance to TAM and could be applied to ER- breast cancer. Although the combination of SK and TAM has been studied, including promotion of the anti-estrogen effect of TAM and modulation of PI3K/AKT pathway, the bypassing of cancer drug resistance by targeting the “weak point” of cancer has not been investigated further.

In the present study, MCF-7 (ER +) and MDA-MB-435S (ER-) cells were used to explore the auxiliary effect of SK to 4-OHT (an active metabolite of TAM in vivo) focusing on cell apoptosis. The optimum concentrations of SK and 4-OHT were determined through CCK-8 assay. The results of comparison and analysis showed that 4-OHT caused a more potent inhibition of MCF-7 than MDA-MB-435S, which was in accordance with its property as an ER + inhibitor. By contrast, SK exhibited more potent inhibition on MDA-MB-435S than MCF-7, which was also consistent with its toxicity toward drug-sensitive and resistant breast cancer cells. The synergistic effects of SK and 4-OHT on MCF-7 cell were considerably better than on MDA-MB-435S cell. This result is attribute to SK’s inhibition of the growth of estrogen-dependent tumor cells and promotion of the anti-estrogen effect of 4-OHT. The following cell apoptosis results showed that SK and 4-OHT had significant synergistic effects on increasing apoptosis in MCF-7 and MDA-MB-435S cells. However, the combination treatment remarkably increased the percentage of early apoptotic cells in the MCF-7 cell and the percentage of late apoptotic cells in the MDA-MB-435S cell. This difference is consistent with their effects on the changing mitochondrial membrane potential. The synergistic effects of SK and 4-OHT on decreasing mitochondrial membrane potential in MCF-7 cell were more significant than on that in MDA-MB-435S cell. Finally, the in vivo experiment indicated that SK also exhibited synergistic effects with 4-OHT on the suppression of tumor growth. 4-OHT can reduce the toxic side effects of SK dramatically, but this result requires further investigation.

This work has some limitations. First, only two types of cell lines were used in the experiment. More types of breast cancer cell lines could be taken into account, including the TAM-resistant cell line, MCF-7R. We also investigated the combined effects of SK and 4-OHT on MDA-MB-231 before (Additional file 1: Table S4 and Figure S1). However, their combined effects on MDA-MB-231 cells were simple additive or even antagonistic. The combination of SK and 4-OHT may trigger different reactions on different types of cells. This combination could bypass drug resistance by inducing necroptosis and apoptosis. However, programmed cell death has been updated and expanded to 12 types in the last 20 years, and the barriers between them are not strict. Thus, other mechanisms of programmed cell death induced by the combined therapy cannot be ruled out. Third, the in vivo study lacked survival data because all mice were alive after six treatments. The combined effects of SK and 4-OHT on MDA-MB-435S xenograft model were not studied due to the low tumor formation rate of MDA-MB-435S cells. As such, additional preclinical data are needed for the clinical applications of SK.

As to the efficiency of Chinese herbal medicine, there still exists a controversial debate to some extent due to its scientific nature of Chinese medicinal ways unlike western medical ways. Undoubtedly, Chinese herbal medicine might be more effective as one disease shows more complex, especially for some complicated and chronic diseases with unknown or complex pathophysiology, such as Alzheimer’s disease, which caused by multiple factors, since such traditional medicine usually contains multiple compounds as a complex with known or unknown multi-functional targets [39, 40]. Another approach for modernization of Chinese herbal medicine is to identify single compound or simple complex with strong medical effect from Chinese traditionally medicinal herbs. Typically, SK is an active natural naphthoquinone compound as one example, which is derived from the dried root of traditional Chinese medicinal herb *Zicao*. Currently, we obtain SK as single compound through modern separation methods, and investigate its anticancer activity with possible underlying mechanism, in order to promote efficiently intracellular delivery with target through modern approaches, and finally intend to develop some effective treatment for human cancer as did in other reports [41], which could be regarded as one attempt of modern experimentations of Chinese traditionally herbal medicine and should be enhanced further for the perspective of Chinese medicine.

## Conclusions

This study demonstrated that combined SK and 4-OHT synergistically inhibited MCF-7 and MDA-MB-435S proliferation in vitro by increasing ROS, decreasing mitochondrial potential, and inducing cell apoptosis mainly through mitochondrial-dependent apoptosis pathway. The combined therapy works in vivo. Therefore, SK may be a potent adjuvant agent to 4-OHT by preventing TAM- resistance in ER + breast cancer treatment and expanding the use of 4-OHT in ER − breast cancer treatment.

## Supplementary information

**Additional file 1: Table S1.** Inhibition of MCF-7 cell proliferation and Q value by the treatment of SK, 4-OHT, and the combination. **Table S2.** Inhibition of MDA-MB-435S cell proliferation and Q value by the treatment of SK, 4-OHT, and the combination. **Table S3.** Tumor growth inhibition rate in mice. **Table S4.** Inhibition of MDA-MB-231 cell proliferation and Q value by the treatment of SK, 4-OHT, and the combination. **Figure S1.** Effects of SK, 4-OHT, and the combination on MDA-MB-231 cell proliferation. **Figure S2.**^1^H NMR of Shikonin. **Figure S3.**^13^C NMR of Shikonin. **Figure S4.** HPLC of Shikonin. **Figure S5.** Circular dichroism spectrum of Shikonin. **Figure S6.** Mass spectrum of Shikonin.

## Data Availability

The datasets used in this study are available from the corresponding author upon reasonable request.

## Abbreviations

4-OHT: 4-Hydroxytamoxifen
ER+: Estrogen receptor positive
SK: Shikonin
ROS: Reaction oxygen species
TAM: Tamoxifen
ER: Estrogen receptor negative
MMP: Metalloprotease
PKM2: pyruvate kinase-M2
CCK-8: Cell Counting Kit-8
PI: Propidium Iodide
PBS: Phosphate buffer saline
