# Supplementary material for: Shikonin and 4-hydroxytamoxifen synergistically inhibit the proliferation of breast cancer cells through activating apoptosis signaling pathway in vitro and in vivo
Source: Chin Med. 2020 Mar 10;15:23. doi: 10.1186/s13020-020-00305-1 (PMC7063777; doi:10.1186/s13020-020-00305-1)
Supplement: Supplementary file 1 — Additional file 1: Table S1. Inhibition of MCF-7 cell proliferation and Q value by the treatment of SK, 4-OHT, and the combination. Table S2. Inhibition of MDA-MB-435S cell proliferation and Q value by the treatment of SK, 4-OHT, and the combination. Table S3. Tumor growth inhibition rate in mice. Table S4. Inhibition of MDA-MB-231 cell proliferation and Q value by the treatment of SK, 4-OHT, and the combination. Figure S1. Effects of SK, 4-OHT, and the combination on MDA-MB-231 cell proliferation. Figure S2.1H NMR of Shikonin. Figure S3.13C NMR of Shikonin. Figure S4. HPLC of Shikonin. Figure S5. Circular dichroism spectrum of Shikonin. Figure S6. Mass spectrum of Shikonin. [file 13020_2020_305_MOESM1_ESM.docx]

**Supporting Information**

**Shikonin and** **4-hydroxytamoxifen synergistically inhibit the proliferation of breast cancer cells through activating apoptosis signaling pathway in *vitro* and in *vivo***

Hong-Yan Lin^1,2†^, Hong-Wei Han^1,2†^, Yin-Song Wang^1^, De-Liu He^1^, Wen-Xue Sun^1^, Lu Feng^1^, Zhong-Ling Wen^1^, Min-Kai Yang^1^, Gui-Hua Lu^1,3^*, Xiao-Ming Wang^1,2^*, Jin-Liang Qi^1,2^*, Yong-Hua Yang^1,2^*

*^1^State Key Laboratory of Pharmaceutical Biotechnology, Institute of Plant Molecular Biology, School of Life Sciences, Nanjing University, Nanjing 210023, PR China*

*^2^Co-Innovation Center for Sustainable Forestry in Southern China, Nanjing Forestry University, Nanjing, 210037, PR China*

*^3^School of Life Sciences, Huaiyin Normal University, Huaian 223300, China*

**Table S1 Inhibition of MCF-7 cell proliferation and Q value by the treatment of SK, 4-OHT, and the combination.**

| SK | | 4-OHT | | SK+4-OHT | | |
| --- | --- | --- | --- | --- | --- | --- |
| Concentration  (μM) | Inhibition rate | Concentration  (μM) | Inhibition rate | Concentration  (μM) | Inhibition rate | Q value |
| 2 | 0.02±0.003 | 15 | 0.14±0.01 | 2+15 | 0.28±0.01 | 1.78 |
| 3 | 0.12±0.008 | 17.5 | 0.28±0.03 | 2+17.5 | 0.51±0.01 | 1.75 |
| 4 | 0.46±0.03 | 20 | 0.47±0.04 | 2+20 | 0.80±0.03 | 1.66 |
|  |  |  |  | 3+15 | 0.47±0.05 | 1.93 |
|  |  |  |  | 3+17.5 | 0.68±0.05 | 1.88 |
|  |  |  |  | 3+20 | 0.83±0.03 | 1.56 |
|  |  |  |  | 4+15 | 0.75±0.04 | 1.41 |
|  |  |  |  | 4+17.5 | 0.84±0.02 | 1.37 |
|  |  |  |  | 4+20 | 0.88±0.04 | 1.23 |

**Table S2 Inhibition of MDA-MB-435S cell proliferation and Q value by the treatment of SK, 4-OHT, and the combination.**

| SK | | 4-OHT | | SK+4-OHT | | |
| --- | --- | --- | --- | --- | --- | --- |
| Concentration  (μM) | Inhibition rate | Concentration  (μM) | Inhibition rate | Concentration  (μM) | Inhibition rate | Q value |
| 2 | 0.29±0.03 | 15 | 0.04±0.01 | 2+15 | 0.42±0.04 | 1.32 |
| 3 | 0.41±0.02 | 17.5 | 0.14±0.02 | 2+17.5 | 0.52±0.05 | 1.33 |
| 4 | 0.53±0.04 | 20 | 0.32±0.03 | 2+20 | 0.56±0.04 | 1.08 |
|  |  |  |  | 3+15 | 0.52±0.03 | 1.20 |
|  |  |  |  | 3+17.5 | 0.66±0.04 | 1.34 |
|  |  |  |  | 3+20 | 0.68±0.02 | 1.13 |
|  |  |  |  | 4+15 | 0.59±0.06 | 0.99 |
|  |  |  |  | 4+17.5 | 0.74±0.02 | 1.24 |
|  |  |  |  | 4+20 | 0.79±0.02 | 1.16 |

**Table S3 Tumor growth inhibition rate in mice.**

| Groups | Tumor growth inhibition rate |
| --- | --- |
| SK (1.5 mg/kg) | 57.20% |
| 4-OHT (3 mg/kg) | 45.44% |
| SK + 4-OHT | 76.65% |

**Table S4 Inhibition of MDA-MB-231 cell proliferation and Q value by the treatment of SK, 4-OHT, and the combination.**

| SK | | 4-OHT | | SK+4-OHT | | |
| --- | --- | --- | --- | --- | --- | --- |
| Concentration  (μM) | Inhibition rate | Concentration  (μM) | Inhibition rate | Concentration  (μM) | Inhibition rate | Q value |
| 2 | 0.18±0.001 | 15 | 0.05±0.01 | 2+15 | 0.21±0.02 | 0.95 |
| 3 | 0.40±0.03 | 17.5 | 0.10±0.02 | 2+17.5 | 0.29±0.02 | 1.11 |
| 4 | 0.53±0.03 | 20 | 0.18±0.02 | 2+20 | 0.33±0.03 | 1.01 |
|  |  |  |  | 3+15 | 0.40±0.03 | 0.93 |
|  |  |  |  | 3+17.5 | 0.38±0.01 | 0.83 |
|  |  |  |  | 3+20 | 0.50±0.01 | 0.98 |
|  |  |  |  | 4+15 | 0.49±0.05 | 0.88 |
|  |  |  |  | 4+17.5 | 0.55±0.02 | 0.95 |
|  |  |  |  | 4+20 | 0.59±0.06 | 0.96 |


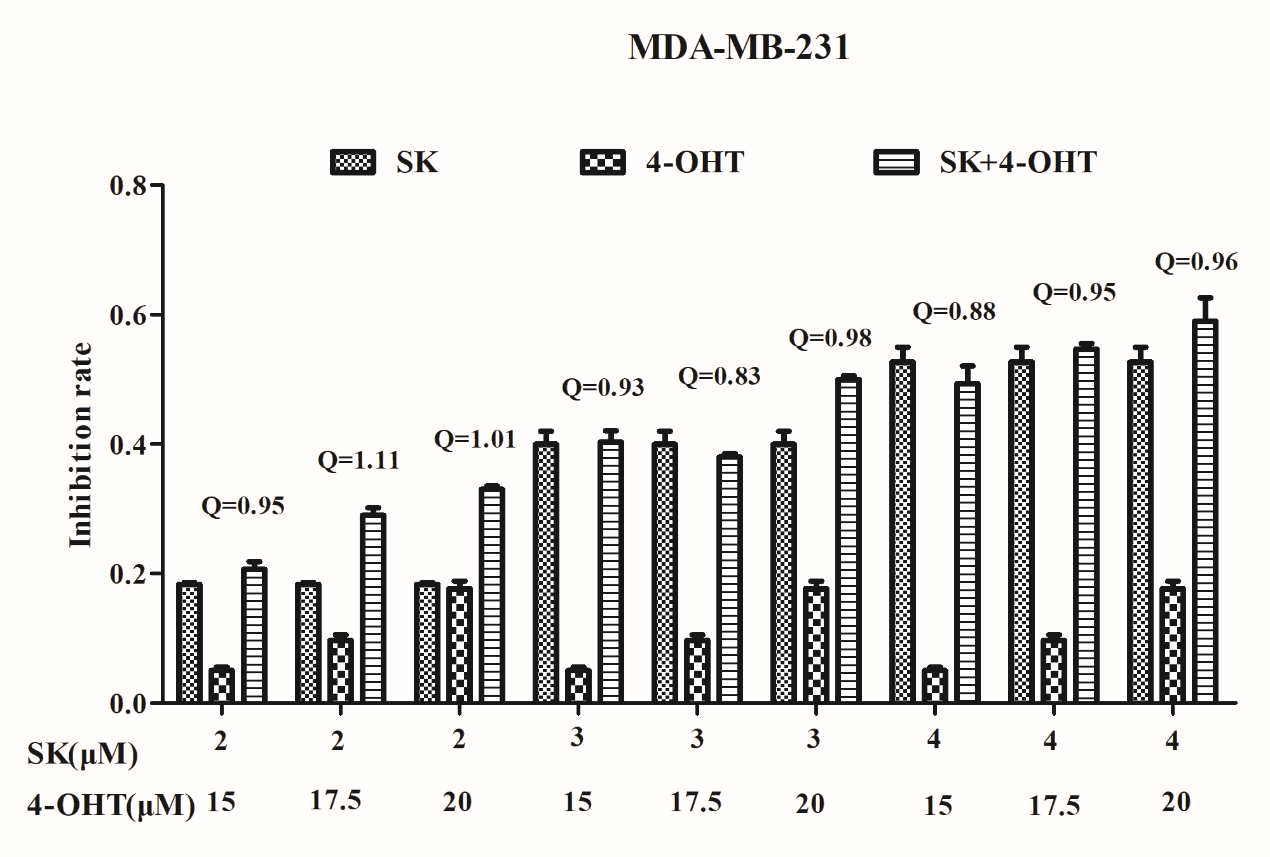


**Fig. S1 Effects of SK, 4-OHT, and the combination on MDA-MB-231 cell proliferation.** The proliferation inhibition rate of MDA-MB-231 cells treated with SK (2, 3, 4 μM), 4-OHT (15, 17.5, 20 μM), or the combination at 24 hours. Q<0.85 suggests antagonism between the two drugs, 0.85≤Q<1.15 addition of their effect, and Q≥1.15 synergism between them. Data are representative of three independent experiments. Each value represents the mean ± S.E.M (n=3).

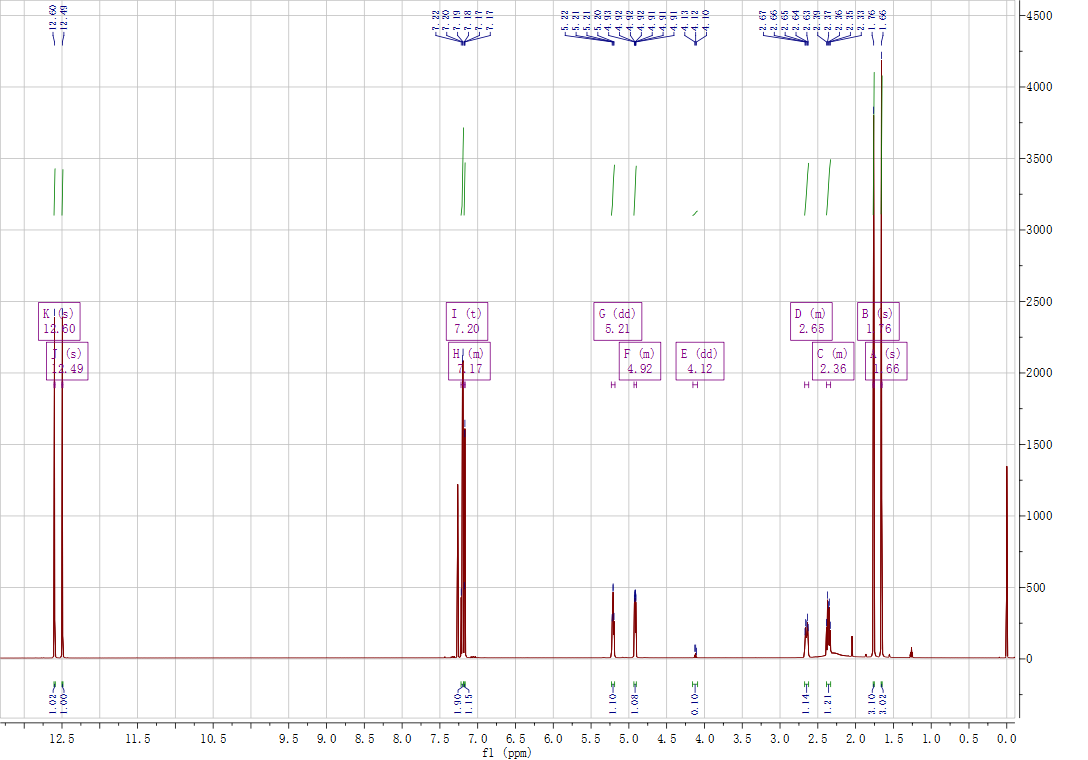


**Fig. S2 ^1^H NMR of Shikonin**


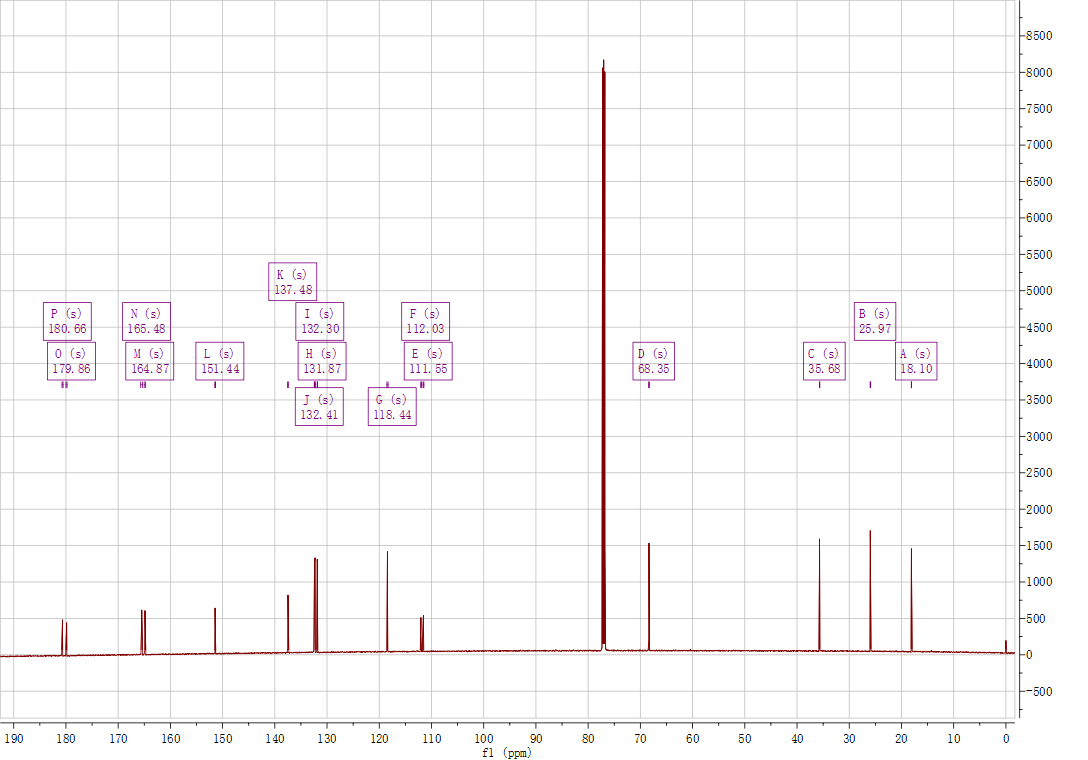


**Fig. S3 ^13^C NMR of Shikonin**

Red powder, Mp: 147-148 °C. ^1^H NMR (600 MHz, CDCl_3_) δ 12.60 (s, 1H, -OH), 12.49 (s, 1H, -OH), 7.20 (t, J = 7.2 Hz, 2H, Ar-H), 7.18 – 7.17 (m, 1H, -C-CH=C), 5.21 (dd, J = 8.0, 6.9 Hz, 1H, -OH), 4.93 – 4.90 (m, 1H, -C-CH=C), 4.12 (dd, J = 14.3, 7.1 Hz, 1H, -O-CH), 2.67 – 2.62 (m, 1H, -CH_2_), 2.39 – 2.33 (m, 1H, -CH_2_), 1.76 (s, 3H, -CH_3_), 1.66 (s, 3H, -CH_3_). ^13^C NMR (151 MHz, CDCl_3_) δ 180.66 (s, 8C), 179.86 (s, 5C), 165.48 (s, 4C), 164.87 (s, 1C), 151.44 (s, 7C), 137.48 (s, 6C), 132.41 (s, 14C), 132.30 (s, 2C), 131.87 (s, 3C), 118.44 (s,13C), 112.03 (s, 9C), 111.55 (s, 10C), 68.35 (s, 11C), 35.68 (s, 12C), 25.97 (s, 15C), 18.10 (s, 16C). ESI-TOF, calcd for C_16_H_16_O_5_ ([M+H]^+^), 289.10, found 288.78. Anal. Calcd for C_16_H_16_O_5_: C, 66.66; H, 5.59; O, 27.75; Found: C, 66.65; H, 5.58; O, 27.72.


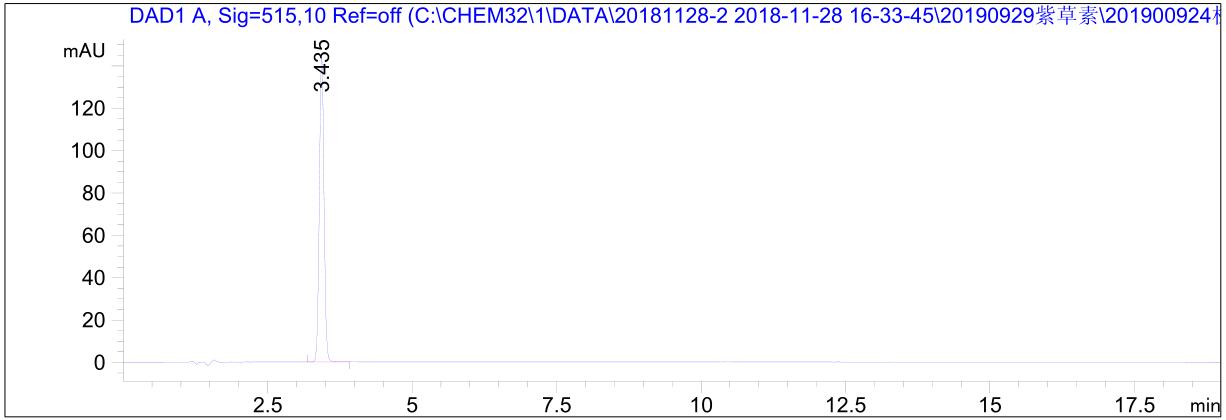


**Fig. S4 HPLC of Shikonin**


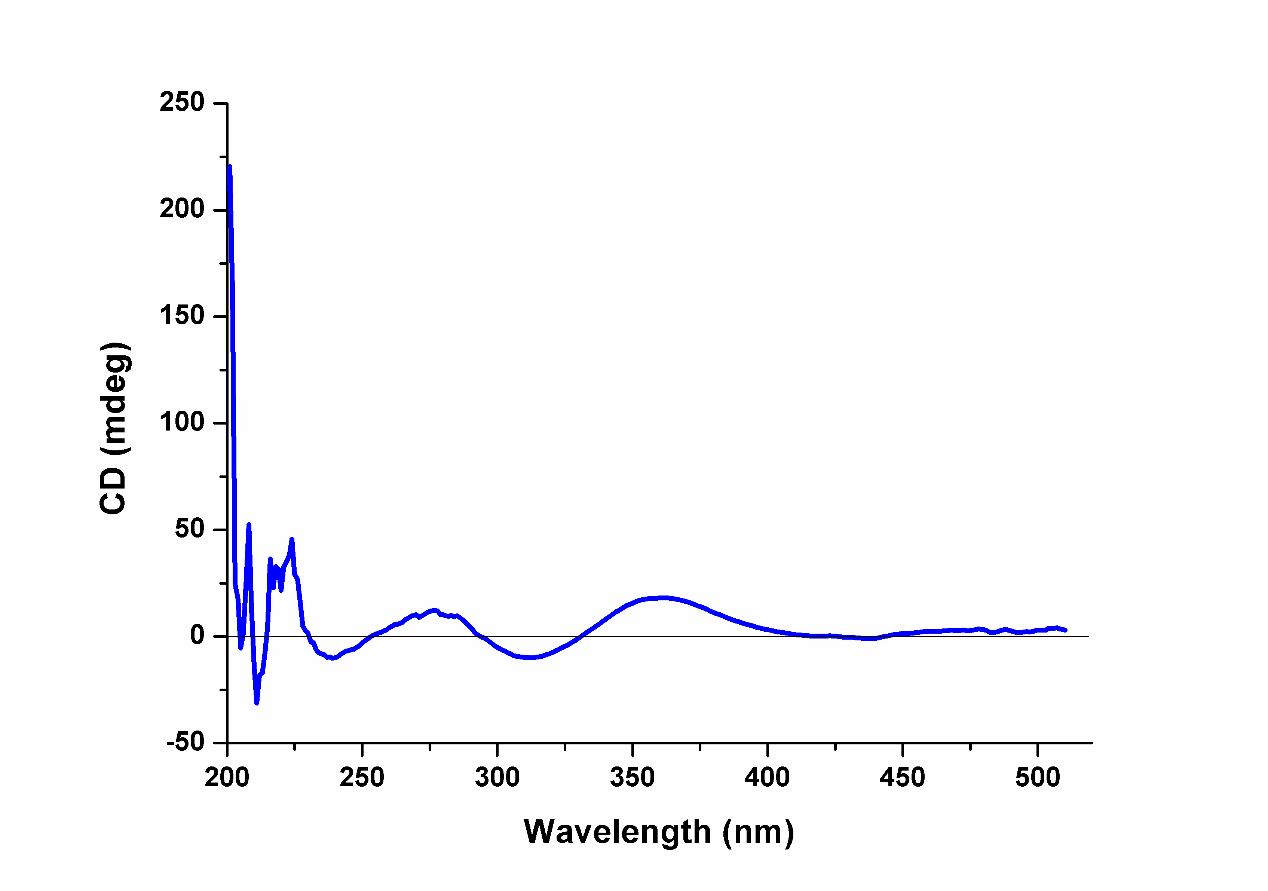


**Fig. S5** **Circular dichroism spectrum of Shikonin**


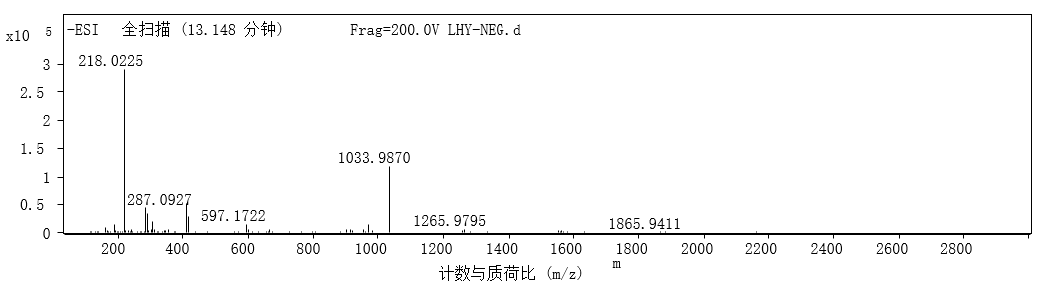


**Fig. S6 Mass spectrum of Shikonin**
